# Comparative Mitochondrial Genome Analyses of Sesarmid and Other Brachyuran Crabs Reveal Gene Rearrangements and Phylogeny

**DOI:** 10.3389/fgene.2020.536640

**Published:** 2020-11-02

**Authors:** Yue-Tian Li, Zhao-Zhe Xin, Ying-Yu Tang, Ting-Ting Yang, Bo-Ping Tang, Yue Sun, Dai-Zhen Zhang, Chun-Lin Zhou, Qiu-Ning Liu, Xiao-Min Yu

**Affiliations:** ^1^Jiangsu Key Laboratory for Bioresources of Saline Soils, Jiangsu Provincial Key Laboratory of Coastal Wetland Bioresources and Environmental Protection, Jiangsu Synthetic Innovation Center for Coastal Bio-agriculture, School of Wetland, Yancheng Teachers University, Yancheng, China; ^2^School of Pharmaceutical Sciences, Wenzhou Medical University, Wenzhou, China; ^3^Key Laboratory of Exploration and Utilization of Aquatic Genetic Resources, College of Aquaculture and Life Science, Shanghai Ocean University, Shanghai, China; ^4^College of Life Sciences, Nankai University, Tianjin, China; ^5^College of Biotechnology and Pharmaceutical Engineering, Nanjing University of Technology, Nanjing, China

**Keywords:** mitochondrial genomes, phylogeny, gene order, crustacean, *Perisesarma bidens*

## Abstract

Mitochondrial genomes (mitogenomes) are important for understanding molecular evolution and phylogenetic relationships. The complete mitogenome of *Perisesarma bidens* was determined, which is 15,641 bp in length. The A + T content of *P. bidens* mitogenome was 74.81%. The AT skew was slightly negative (−0.021). The 22 tRNAs ranged from 65 to 73 bp and were highly A + T biased. All tRNA genes had typical cloverleaf structures, except for the *trnS1* gene, which lacked a dihydrouridine (DHU) arm. The gene order within the *P*. *bidens* mitogenome was identical to the pancrustacean ground pattern, except for the translocation of the *trnH*. Additionally, the gene order of *trnI-trnQ-trnM* in pancrustacean ground pattern became *trnQ-trnI-trnM* in *P. bidens*. Phylogenetic analyses supported the inclusion of *P*. *bidens* in Sesarmidae and the promotion of Sesarminae to Sesarmidae. The results will help us to better understand the status and evolutionary history of Grapsoidea crabs.

## Introduction

Decapoda is the most diverse, species-rich group of crustaceans, containing many well-known animals, such as crayfish, lobsters, shrimps, hermit crabs, and “true” crabs (Shen et al., 2013; Basso et al., 2017). The true crabs belong to Brachyura, which is a diverse, economically important group, with about 7200 described species (De Grave et al., 2009; Ahyong et al., 2011). Brachyura is highly adaptable and can live on land and in both marine and fresh water. Therefore, crabs have become important groups for the study of evolution (Castro et al., 2015). Some Brachyura are edible and medicinal and have economic importance (Carpenter and Niem, 1998).

Most Brachyura are grouped into the Podotremata, Heterotremata, and Thoracotremata, with the latter two referred to as the Eubrachyura. However, the phylogenetic relationships within Eubrachyura remain controversial, particularly the relationships of the Sesarmidae and Varunidae, and between these two and Grapsoidea (Schubart et al., 2000, 2002; Kitaura et al., 2002). The traditional classification of Grapsidae contains four subfamilies: Grapsinae, Plagusiinae, Sesarminae, and Varuninae (Schubart et al., 2000). Traditional methods place the following in the Sesarminae: *Perisesarma bidens*, *Sesarmops sinensis*, *Clistocoeloma sinensis*, *Helice tientsinensis*, *Helice latimera*, *Helice wuana*, and *Metaplax longipes*. Of these, *P. bidens* and *S. sinensis* should be *Sesarmops* crabs; *C*. *sinensis* should be a *Clistocoeloma* crab; *H*. *tientsinensis*, *H*. *latimera*, and *H*. *wuana* should be *Helice* crabs and *M*. *longipes* should be a *Metaplax* crab (Schubart et al., 2000). However, some scholars have suggested that Grapsidae should be promoted to Grapsoidea, promoting the four subfamilies to families, i.e., Grapsidae, Varunidae, Sesarmidae and Plagusiidae. Other studies have advised that *Sesarmops* and *Clistocoeloma* crabs, which originally belonged to Sesarminae, should belong to Sesarmidae, and that *Helice* and *Metaplax* crabs that originally belonged to Sesarminae should be transferred from the Sesarminae to the Varunidae (Kitaura et al., 2002; Schubart et al., 2002). The classification of these taxa remains unresolved. Sesarmid crabs are common in mangroves and can tolerate great variation in salinity along the environment (Theuerkauff et al., 2018). They are very good experimental research objects.

Many studies have investigated these relationships using nuclear DNA, mitochondrial DNA (mtDNA), and morphological character analyses. Some studies combined mtDNA and nuclear genes to reconstruct more reliable phylogenetic trees. However, the combination of these genes made alignment and model selection quite difficult (Foster, 2004; Cox et al., 2008). This has led to the conclusion that the taxon sampling is insufficient and unbalanced (Bergsten, 2005; Wägele and Mayer, 2007). It is evident that more species are necessary to improve the quality of the analyses and stability of phylogenetic trees (Brinkmann and Philippe, 2008).

The mitochondrial genome (mitogenome) has been widely used in phylogenetic analyses, due to its rich signals from sequence information and gene arrangement (Xin et al., 2017a, b). The mitogenome has a simple structure, haploid nature, maternal inheritance, and rapid evolutionary rate (Liu et al., 2015). The mitogenomes are closed circular double-stranded molecules in the range of 14–18 kb in most bilaterian animals, including 13 protein-coding genes (PCGs; *cox1–3*, *cob*, *nad1–6* and *nad4L*, *atp6*, and *atp8*), 2 rRNA genes, 22 tRNA genes, and an AT-rich region (control region) (Tang et al., 2003, 2017, 2018; Xin et al., 2017a, b). The taxonomy of Sesarmid crabs has been studied extensively and benefited from recent refinements in species of *Perisesarma* and *Sesarmops* (Li et al., 2019; Shih et al., 2019; Ng et al., 2020). However, the mitogenome of *P. bidens* has not been analyzed. Here, we determined the mitogenome of *P. bidens* and used the mitogenomes of 65 species to construct phylogenetic trees to discuss the systemic status and genetic relationships of the controversial taxa, Sesarmidae and Grapsoidea.

## Materials and Methods

### Ethics Statement

We have taken a close look at the website^1^. We found that the species *Perisesarma bidens* is not considered endangered or protected species, the IUCN status for this species is “Not evaluated.” Similarly, the species *Perisesarma bidens* is also Not endangered or protected species in China. No special permit Is required to collect crabs at selected sites in China. the sampling locations are Not privately-owned or natural protected areas, the collection of this species is legal in China. So we can use this species for experiments and subsequent analysis.

### Sample Collection

Specimens of *P*. *bidens* were collected from the seaside of Zhangzhou City, Fujian Province, China, identified using the morphological methods of Dai (1999) and molecular identification with COI marker, and preserved in 95% ethanol at –20°C until DNA extraction. Voucher specimens of *P*. *bidens* were deposited in the Jiangsu Provincial Key Laboratory of Coastal Wetland Bioresources and Environmental Protection, School of Ocean and Biological Engineering, Yancheng Teachers University, Yancheng, China.

### DNA Extraction, PCR, and Genome Sequencing

Total genomic DNA was extracted from muscle using a genomic DNA extraction kit (Sangon, China), following the manufacturer’s instructions, and was visualized on 1.0% agarose gels. The complete mitogenome was obtained using a combination of conventional PCR and long PCR to amplify overlapping fragments spanning the entire mitogenome. Initially, conserved sequences, such as *cox1*, *cox3*, *nad5*, *nad4*, and *rrnS*, were amplified by conventional PCR using universal primers synthesized by Beijing Sunbiotech (Tang et al., 2003, 2017, 2018; Liu et al., 2015; Xin et al., 2017a, b).

We designed species-specific primers to amplify large overlapping regions of the mitogenome based on conserved sequences using Primer Premier 5 (Supplementary Table S1). All amplifications were performed on a Mastercycler (Eppendorf) and Mastercycler gradient. The reactions were 50 μL and contained 34.65 μL ddH_2_O, 5 μL 10 × LA PCR buffer II (Mg^2+^ Plus, Aidlab), 4 μL dNTPs (10 mM), 2 μL each primer (10 μM), 0.35 μL red *Taq* DNA Polymerase (5 U/μL, Aidlab), and 2 μL DNA template (∼30 ng).

The PCR conditions for conserved sequences followed a standard three-step protocol, with an initial denaturing at 96°C for 3 min, then 40 cycles of 94°C for 30 s, annealing at the recommended temperature for each primer for 30 s, and elongation at 72°C for 45 s, with a final 5 min extension at 72°C. The PCR conditions for large overlapping regions followed a standard two-step protocol with 3 min at 94°C, followed by 35 cycles of 35 s at 94°C, 3–6 min at 50–56°C, and 10 min at 72°C. All PCR products were sent to General Biosystems, Anhui for Sanger sequencing.

### Annotation and Alignment

The sequence was annotated using DNASTAR (DNASTAR, Madison, WI, United States). The locations of the PCGs, rRNA genes, tRNA genes, and CR were initially identified using the MITOS Web Server^2^. The PCG coding regions were further identified using the NCBI ORF Finder^3^. Two rRNA genes were identified by alignment with published brachyuran sequences. Codon usage and the nucleotide composition of the mitogenomes were determined using MEGA6 (Tamura et al., 2013). The nucleotide sequence of the complete *P*. *bidens* mitogenome was deposited in the NCBI database under accession no. KY808394. Gene orders in the complete mitogenome were also inferred through the MITOS Web Server.

### Phylogenetic Analyses

We used nucleotide (NT) sequences for phylogenetic analyses. The sequences were aligned with MAFFT using the default settings (Katoh et al., 2002). Gaps in the sequences were removed using Gblocks (Castresana, 2000), and the saturation of the sequences was examined using DAMBE (Xia and Xie, 2001), which indicated that the sequences were not saturated and were suitable for phylogenetic analyses. Complete mitogenomes of 65 decapods (60 crabs plus 5 outgroups) were downloaded from NCBI (Table 1). The five outgroups were *Cherax destructor*, *Cambaroides similis*, *Neopetrolisthes maculatus*, *Paralithodes camtschaticus*, and *Pagurus longicarpus*.

**TABLE 1:** List of brachyuran species with their GenBank accession numbers.

Species	Family	Superfamily	Size (bp)	Accession No.
*Sesarmops sinensis*	Sesarmidae	Grapsoidea	15,905	KR336554
*Clistocoeloma sinensis*	Sesarmidae		15,706	KU589292
*Perisesarma bidens*	Sesarmidae		15,641	KY808394
*Metaplax longipes*	Varunidae		16,424	MF198248
*Helice latimera*	Varunidae		16,246	KU589291
*Helice tientsinensis*	Varunidae		16,212	KR336555
*Helice wuana*	Varunidae		16,359	KX344898
*Sesarma neglectum*	Sesarmidae		15,920	KX156954
*Metopaulias depressus*	Sesarmidae		15,765	KX118277
*Parasesarmops tripectinis*	Sesarmidae		15,612	KU343209
*Eriocheir japonica japonica*	Varunidae		16,352	FJ455505
*Eriocheir japonica sinensis*	Varunidae		16,378	KM516908
*Eriocheir japonica hepuensis*	Varunidae		16,335	FJ455506
*Cyclograpsus granulosus*	Varunidae		16,300	LN624373
*Pachygrapsus crassipes*	Grapsidae		15,652	KC878511
*Grapsus tenuicrustatus*	Grapsidae		15,858	KT878721
*Xenograpsus testudinatus*	Xenograpsidae		15,798	EU727203
*Xenograpsus ngatama*	Xenograpsidae		16,106	KY985236
*Portunus pelagicus*	Portunidae	Portunoidea	16,157	KM977882
*Callinectes sapidus*	Portunidae		16,263	AY363392
*Portunus trituberculatus*	Portunidae		16,026	AB093006
*Portunus sanguinolentus*	Portunidae		16,024	KT438509
*Charybdis japonica*	Portunidae		15,738	FJ460517
*Scylla paramamosain*	Portunidae		15,824	JX457150
*Scylla olivacea*	Portunidae		15,723	FJ827760
*Scylla tranquebarica*	Portunidae		15,833	FJ827759
*Scylla serrata*	Portunidae		15,775	FJ827758
*Charybdis feriata*	Portunidae		15,660	KF386147
*Charybdis natator*	Portunidae		15,664	MF285241
*Thalamita crenata*	Portunidae		15,787	LK391945
*Chaceon granulatus*	Geryonidae		16,135	AB769383
*Chaceon* sp.	Geryonidae		16,126	KU507298
*Gandalfus yunohana*	Bythograeidae	Bythograeoidea	15,567	EU647222
*Gandalfus puia*	Bythograeidae		15,548	KR002727
*Austinograea alayseae*	Bythograeidae		15,620	JQ035660
*Austinograea rodriguezensis*	Bythograeidae		15,611	JQ035658
*Segonzacia mesatlantica*	Bythograeidae		15,521	KY541839
*Homologenus malayensis*	Homolidae	Homoloidea	15,793	KJ612407
*Moloha majora*	Homolidae		15,903	KT182069
*Geothelphusa dehaani*	Potamidae	Potamoidea	18,197	AB187570
*Longpotamon xiushuiense*	Potamidae		18,460	KU042041
*Huananpotamon lichuanense*	Potamidae		15,380	KX639824
*Somanniathelphusa boyangensis*	Parathelphusidae		17,032	KU042042
*Pseudocarcinus gigas*	Eriphiidae	Xanthoidea	15,515	AY562127
*Leptodius sanguineus*	Xanthidae		15,480	KT896744
*Myomenippe fornasinii*	Menippidae	Eriphioidea	15,658	LK391943
*Ocypode cordimanus*	Ocypodidae	Ocypodoidea	15,604	KT896743
*Ocypode ceratophthalmus*	Ocypodidae		15,564	LN611669
*Ilyoplax deschampsi*	Dotillidae		15,460	JF909979
*Mictyris longicarpus*	Mictyridae		15,548	LN611670
*Macrophthalmus japonicus*	Macrophthalmidae		16,170	KU343211
*Umalia orientalis*	Raninidae	Raninoidea	15,466	KM365084
*Lyreidus brevifrons*	Raninidae		16,112	KM983394
*Ranina ranina*	Raninidae		15,563	KM189817
*Dynomene pilumnoides*	Dynomenidae	Dromioidea	16,475	KT182070
*Ashtoret lunaris*	Matutidae	Calappoidea	15,807	LK391941
*Maja squinado*	Majidae	Majoidea	16,598	KY650652
*Maja crispata*	Majidae		16,592	KY650651
*Chionoecetes japonicus*	Majidae		15,341	AB735678
*Damithrax spinosissimus*	Mithracidae		15,817	KM405516
*Cherax destructor*	Parastacidae	Parastacoidea	15,713	HG799087
*Cambaroides similis*	Cambaridae	Astacoidea	16,220	NC016925
*Neopetrolisthes maculatus*	Porcellanidae	Galatheoidea	15,324	KC107816
*paralithoeds camtschaticus*	Lithodidae	Paguroidea	16,720	NC020029
*pagurus longicarpus*	Paguridae		15,630	AF150756

Phylogenetic analyses were performed using Bayesian inference (BI) and maximum likelihood (ML) methods using MrBayes v 3.2.2 (Ronquist et al., 2012) and IQ-Tree (Nguyen et al., 2014; Kalyaanamoorthy et al., 2017; Hoang et al., 2018), respectively. The GTRmodel was selected by MrModeltest 2.3 (Nylander, 2004). The BI analyses ran four independent chains for 10,000,000 generations, sampled every 100 generations, with a burn-in of 25,000 generations. The average standard deviation of split frequencies was < 0.01. Convergence was assessed using Tracer v1.6 and the effective sampling size for all parameters was > 200. The ML analyses were performed on 1000 bootstrap replications. The resulting phylogenetic trees were visualized using FigTree v1.4.2.

## Results and Discussion

### Genome Structure, Organization, and Composition

**FIGURE 1:**
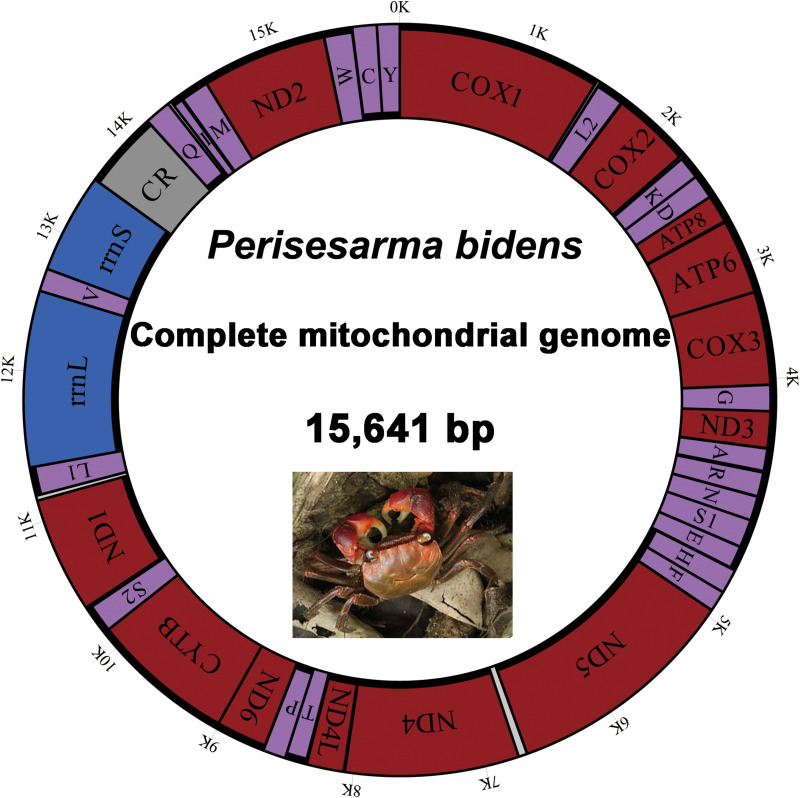
Map of the *P*. *bidens* mitogenome. Protein-coding and ribosomal genes are shown with standard abbreviations. Genes for tRNAs are abbreviated by single letters, with S1 = AGN, S2 = UCN, L1 = CUN, and L2 = UUR. CR, control region.

**FIGURE 2:**
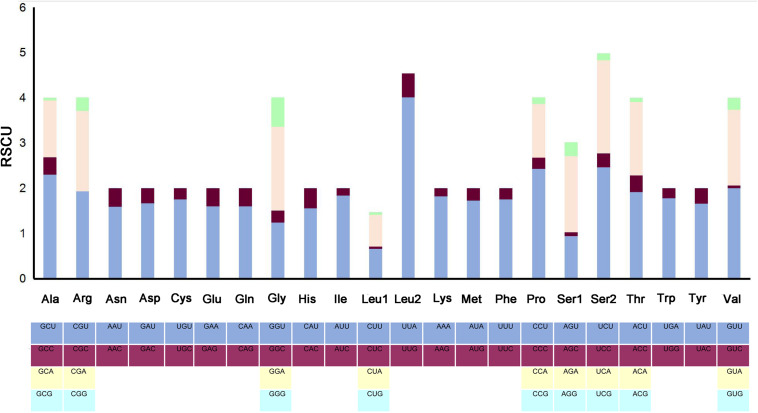
The RSCU in the *P*. *bidens* mitogenome.

**FIGURE 3:**
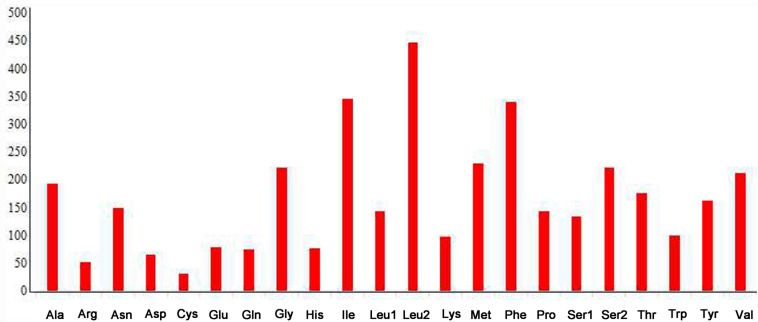
Amino acid composition of the *P*. *bidens* mitogenome.

The complete mitogenome of *P*. *bidens* was a circular of 15,641 bp (GenBank accession no. KY808394). Its size was within the range observed in completely sequenced brachyuran species. The mitogenome composition (A: 36.61%, T: 38.20%, C: 15.13%, G: 10.06%) was strongly A + T biased which accounts for 74.81%, and exhibited with negative AT-skew (–0.021). The AT-skew of the mitogenomes of most crabs were negative, for example, *H*. *wuana* (Tang et al., 2018), *S. sinensis* (Tang et al., 2017), *H*. *tientsinensis* (Xin et al., 2017b), *C*. *sinensis* (Xin et al., 2017a), the AT-skew value of mitogenomes in other crabs had also been calculated and counted in related studies (Xin et al., 2017a, b). The genes were typical of animal mitogenomes, with 22 tRNA genes, 13 PCGs, 2 rRNA genes, and a CR (Table 2). Overall, 4 of the 13 PCGs (*nad5*, *nad4*, *nad4L*, and *nad1*), 8 tRNAs [*trnQ*, *trnC*, *trnY*, *trnF*, *trnH*, *trnP*, *trnL* (CUN), and *trnV*], and 2 rRNAs (*rrnL* and *rrnS*) were encoded by the minority strand, while the other 23 genes were encoded by the majority strand (Table 2 and Figure 1). The 13 PCGs ranged from 159 to 1731 bp. Of 22 tRNA genes, 8 were encoded by the L-strand and the remaining 14 by the H-strand. All tRNAs had the typical clover-leaf secondary structures observed in mitochondrial tRNA genes, except for *trnS1* (AGN), which lacked a stable dihydrouridine (DHU) arm; this has been observed in several animals, including insect and brachyuran mitogenomes (Liu et al., 2015; Xin et al., 2017a, b). Figure 2 shows the relative synonymous codon usage (RSCU) of *P*. *bidens*. The codon usage was biased with a high frequency of AT compared to GC in the third codon position. The codon usage analysis revealed that the leucine 2 (*Leu2*), isoleucine (*Ile*), phenylalanine (*Phe*) codon families were most frequently utilized, while cysteine (*Cys*) family was the least used (Figure 3).

**TABLE 2:** Summary of the *P*. *bidens* mitogenome.

Gene	Direction	Location	Size (bp)	Intergenic nucleotides
*cox1*	F	1–1560	1560	−25
*trnL2*	F	1536–1604	69	5
*cox2*	F	1610–2317	708	−20
*trnK*	F	2298–2366	69	0
*trnD*	F	2367–2434	68	0
*atp8*	F	2435–2593	159	−7
*atp6*	F	2587–3261	675	−1
*cox3*	F	3261–4052	792	−1
*trnG*	F	4052–4116	65	0
*nad3*	F	4117–4467	351	2
*trnA*	F	4470–4536	67	10
*trnR*	F	4547–4612	66	2
*trnN*	F	4615–4681	67	0
*trnS1*	F	4682–4748	67	1
*trnE*	F	4750–4815	66	4
*trnH*	R	4820–4884	65	0
*trnF*	R	4885–4950	66	1
*nad5*	R	4952–6682	1731	41
*nad4*	R	6742–8073	1350	−7
*nad4L*	R	8067–8369	303	8
*trnT*	F	8378–8443	66	0
*trnP*	R	8444–8509	66	2
*nad6*	F	8512–9015	504	−1
*cob*	F	9015–10,149	1135	0
*trnS2*	F	10,150–10,217	68	15
*nad1*	R	10,233–11,171	939	34
*trnL1*	R	11,206–11,271	66	0
*rrnL*	R	11,272–12,612	1341	0
*trnV*	R	12,613–12,685	73	0
*rrnS*	R	12,686–13,515	830	0
CR	—	13,516–14,193	678	0
*trnQ*	R	14,194–14,263	70	23
*trnI*	F	14,287–14,354	68	8
*trnM*	F	14,363–14,431	69	0
*nad2*	F	14,432–15,439	1008	2
*trnW*	F	15,442–15,511	70	−3
*trnC*	R	15,509–15,573	65	0
*trnY*	R	15,574–15,641	68	–

### Gene Order in Sesarmidae

**FIGURE 4:**
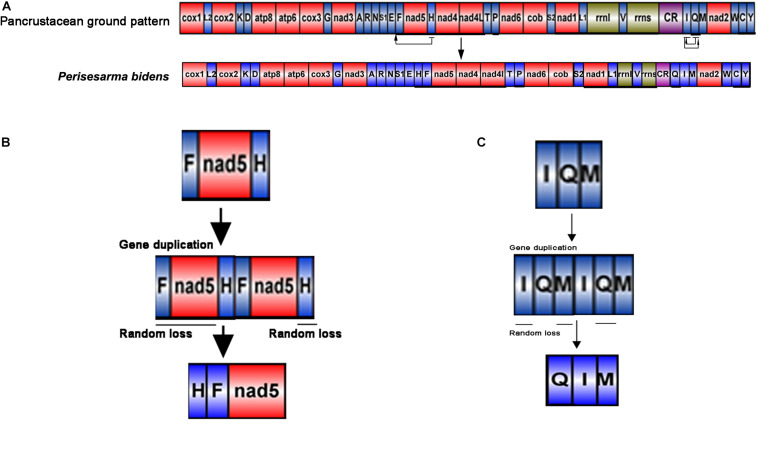
Generation of the *P*. *bidens* mitochondrial gene arrangement. The duplication/random loss, recombination, and duplication/non-random loss models were used to explain the principle of gene rearrangement. **(A)** Comparison of gene order in mitogenome of Perisesarma bidens and Pancrustacean ground pattern. tRNA genes are indicated by the singer letter IUPAC-IUB abbreviation with S1 = AGN, S2 = UCN, L1 = CUN, and L2 = UUR, where as protein and rRNA genes are labled with three letter codes. **(B)** Gene duplication occurred in *trnF*, *nad5*, and *trnH*, changing the arrangement of *trnF*-*nad5*-*trnH* to *trnF*-*nad5*-*trnH* -*trnF*-*nad5*-*trnH*. Then, the redundant *trnF*, *nad5*, and *trnH* genes were lost at random. Finally, the new gene order of *trnH*-*trnF*-*nad5* was formed. **(C)** Gene duplication occurred in *trnI*, *trnQ*, and *trnM*, changing the arrangement of *trnI*-*trnQ*-*trnM* to *trnI*-*trnQ*-*trnM*-*trnI*-*trnQ*-*trnM*. Then, the redundant *trnI*, *trnM*, and *trnQ* genes were lost at random. Finally, the new gene order of *trnQ*-*trnI*-*trnM* was formed.

The gene order of *P. bidens* was identical to other Sesarmidae species in our study. In contrast to the inferred ancestral gene sequences of Pancrustaceans, where *trnH* was located between *nad5* and *nad4*, here it was found between *trnE* and *trnF*. In Pancrustaceans, the tRNA gene sequences between CR and *trnM* was *trnI-trnQ*, but here was *trnQ-trnI* (Figure 4A).

The duplication/random loss model was used to explain the rearrangements seen in Sesarmidae (Moritz and Brown, 1987; Macey et al., 1997; Boore and Brown, 1998). The movement of *trnH* can be explained as follows. First, gene duplication occurred in *trnF*, *nad5*, and *trnH*, changing the arrangement of *trnF-nad5-trnH* to *trnF-nad5-trnH-trnF-nad5-trnH*. Then, the redundant *trnF*, *nad5*, and *trnH* genes were lost at random. Finally, the new gene order of *trnH-trnF-nad5* was formed (Figure 4B). The order principles of *trnQ* moving from the junction between *trnI* and *trnM* to between the CR and *trnI* could also be explained similarly (Figure 4C).

### Gene Order of Crabs From Other Families

**FIGURE 5:**
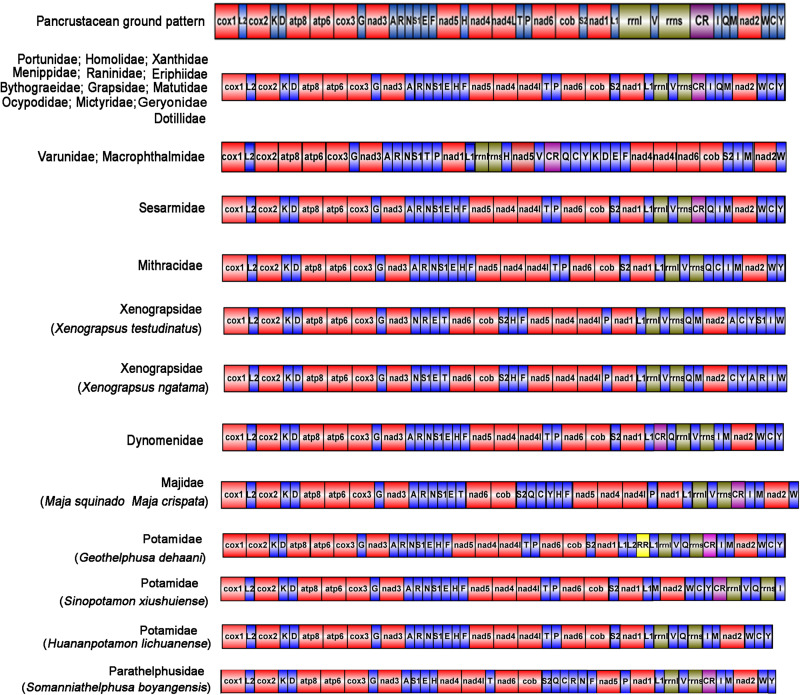
The mitochondrial gene order of all Brachyura.

The gene orders of all species are shown in Figure 5. The gene sequences within 13 families were the same. The gene order pattern of *Macrophthalmus japonicus* (Ocypodoidea, Macrophthalmidae) was identical to that of other Varunidae. The gene orders of *Damithrax spinosissimus* (Majoidea, Mithracidae) and *Dynomene pilumnoides* (Dromioidea, Dynomenidae) were different, as were those of two Xenograpsidae crabs (*Xenograpsus testudinatus* and *X*. *ngatama*). However, two Majidae crabs (*Maja squinado* and *M*. *crispata*) had the same gene order. Interestingly, although there were only four species of Potamoide, each showed a different gene order.

### Phylogenetic Analyses

**FIGURE 6:**
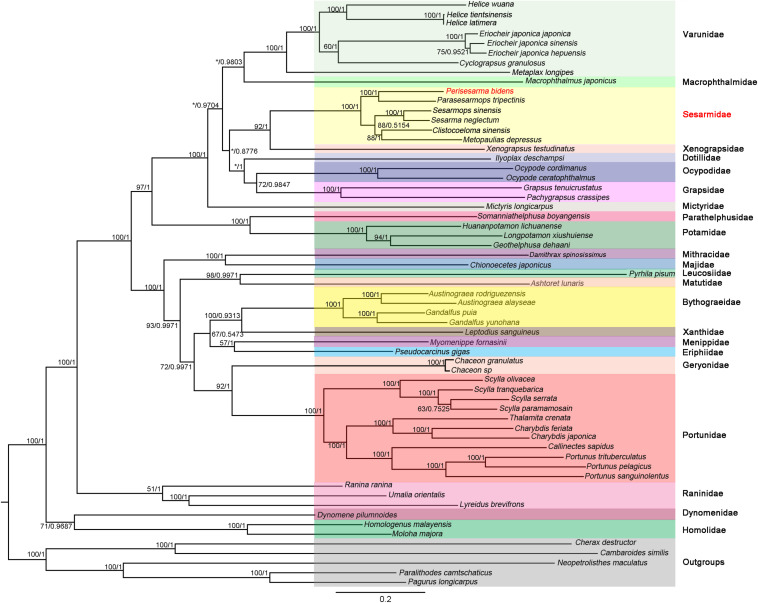
Phylogenetic trees were constructed using BI and ML methods based on NT dataset. Bootstrap values (BP) (IQ-Tree) and Bayesian posterior probability (BPP) of each node are shown as BP based on NT dataset/BPP based on NT dataset. *C*. *destructor*, *C*. *similis*, *N*. *maculatus*, *P*. *camtschaticus*, and *P*. *longicarpus* were used as outgroups. The supermatrix underlying this figure is as a Supplementary File.

The phylogenetic trees were constructed based on 13 PCGs under ML and BI methods, which resulted in congruent tree topologies, except for minor differences within “Grapsoidea + Ocypodoidea” (Figure 6). As shown in Figure 6, *P*. *bidens* formed a well-supported clade with *Parasesarmops tripectinis* (BP = 100; BPP = 1). (*P. bidens* + *P*. *tripectinis*) clade, (*S*. *sinensis* + *S. neglectum*) clade, (*C*. *sinensis* + *M. depressus*) clade were well supported with each other; these results were in accordance with the information provided by the same genes orders of *P. bidens*, *P*. *tripectinis, S. sinensis*, *S*. *neglectum*, *C. sinensis*, and *M*. *depressus.* Moreover, *S*. *neglectum*, *M*. *depressus*, and *P*. *tripectinis* all belonged to Sesarmidae (Park et al., 2018). Therefore, *P*. *bidens*, *S*. *sinensis*, and *C*. *sinensis* should belong to Sesarmidae rather than to Sesarminae. The species originally belonging to the Sesarminae should belong to the Sesarmidae. These results agree with previous analyses using the mitogenome of one species (Tang et al., 2017; Xin et al., 2017a, b).

In our study, two families (Potamidae and Parathelphusidae) were primarily freshwater crabs and were recognized as true heterotremes (Guinot et al., 2013). The systemic status of primary freshwater crabs had stimulated interest because of their high value and diversity (Cumberlidge et al., 2009; Klaus et al., 2010). The monophyly of Potamidae and Parathelphusidae was confirmed based on morphological and molecular analyses. However, there still were uncertainties regarding the phylogenetic placement of Potamidae and Parathelphusidae (Xing et al., 2017). Von Sternberg and Cumberlidge (2001) suggested that these two families Potamidae and Parathelphusidae should be placed in Thoracotremata. Here, the Thoracotremata contained Grapsoidea and Ocypodoidea crabs. Our results showed that four heterotreme crabs (*Geothelphusa dehaani*, *Longpotamon xiushuiense*, *Huananpotamon lichuanense*, and *Somanniathelphusa boyangensis*) were actually more closely associated with thoracotreme crabs, showing that Heterotremata was not monophyletic; this result was in accordance with that inferred from 23 brachyuran crabs, in which the author use the two mitogenomes (Ji et al., 2014). Within Podotremata, the clade was monophyletic. The six crabs formed a robust clade [(Homolidae + Dynomenidae) + Raninidae]. Within Heterotremata, the phylogenetic relationships were clear, with the exception of the four potamid crabs, which were outside of the heterotreme crabs.

## Data Availability Statement

The datasets generated for this study can be found in the GenBank accession no. KY808394.

## Author Contributions

Q-NL, B-PT, and X-MY conceived and designed the study. Z-ZX, Q-NL, Y-YT, and T-TY conducted the molecular work and data analysis. Z-ZX and Y-TL drafted the manuscript. Z-ZX, Q-NL, Y-YT, YS, D-ZZ, C-LZ, and T-TY prepared all figures and tables. Z-ZX and Q-NL performed the phylogenetic analyses. Z-ZX, Q-NL, B-PT, and X-MY contributed to drafting the manuscript. All authors contributed to the article and approved the submitted version.

## Conflict of Interest

The authors declare that the research was conducted in the absence of any commercial or financial relationships that could be construed as a potential conflict of interest.

## Footnotes

1 https://www.gbif.org/en/species/4382775

2 http://mitos.bioinf.uni-leipzig.de/index.py

3 https://www.ncbi.nlm.nih.gov/orffinder/

## Abbreviations

A: adenine
Atp6 and Atp8: genes for the ATPase subunits 6 and 8
BI: Bayesian inference
BP: Base pair
C: cytosine
Cox1-cox3: genes for cytochrome C oxidase subunits I–III
G: guanine
l-rRNA (large): rRNA subunit
Mitogenomes: Mitochondrial genomes
ML: maximum likelihood
mtDNA: mitochondrial DNA
Nad1–nad6 and nad4L: genes for NADH dehydrogenase subunits 1–6 and 4L
PCGs: protein-coding genes
rRNA: ribosomal RNA genes subunit
s-rRNA: (small)
T: thymine
tRNAx: transfer RNA, where X is replaced by three letters amino acid code of the corresponding amino acid.
