# Supplementary material for: Comparative Mitochondrial Genome Analyses of Sesarmid and Other Brachyuran Crabs Reveal Gene Rearrangements and Phylogeny
Source: Front Genet. 2020 Nov 2;11:536640. doi: 10.3389/fgene.2020.536640 (PMC7667018; doi:10.3389/fgene.2020.536640)
Supplement: Supplementary file 1 [file Table_1.docx]

**Table S1**

| Primer | Sequence (5′–3′) | annealing temperature | Location |
| --- | --- | --- | --- |
| F1 | GGTCAACAAATCATAAAGATATTGG | 55℃ | *cox1* |
| R1 | TAAACTTCAGGGTGACCAAAAAATCA |  | *cox1* |
| F2 | TAGTWATHAANGGHCTACGVTGRGG | 50℃ | *cox3* |
| R2 | AAGTCCRTGRAAYCCDGTDGCHAC |  | *cox3* |
| F3 | TATGTGGDWTWCCTTTTWTAGCDGG | 48℃ | *nad5* |
| R3 | ATHTCAAGMTAARCHAGCHCCHCC |  | *nad5* |
| F4 | GTGCCAGCCGCCGCGGTTA | 52℃ | *rrnS* |
| R4 | ATGCACTTTCCAGTACATCTA |  | *rrnS* |
| F5 | TGTTAGACAAGAATCAGGAA | 56℃ | *cox1- cox3* |
| R5 | ATAATAACGTGAAGACCATG |  | *cox1- cox3* |
| F6 | AAGATTGTCACCAACTGTTGAAA | 58℃ | *cox3-nad5* |
| R6 | ACCTTGTTTTGGCTTTCGTATTT |  | *cox3-nad5* |
| F7 | GCTACCGTTATGTAAGCCACCCT | 59℃ | *nad5- rrnS* |
| R7 | TATTTGTGCCAGCATTCGCGGTT |  | *nad5- rrnS* |
| F8 | CATCTACTATGTTACGACTTATT | 56℃ | *rrnS- cox1* |
| R8 | GATAGTCAGAGTAACGCCGAGGT |  | *rrnS- cox1* |
